# Work after cancer-sickness absence. Barriers and facilitators from survivors’ perspective

**DOI:** 10.1093/eurpub/ckac129.312

**Published:** 2022-10-25

**Authors:** A Ayala, L Serra, D Rodriguez-Arjona, FG Benavides

**Affiliations:** 1 Centre for Research in Occupational Health, Universitat Pompeu Fabra, Barcelona, Spain; 2 CIBER of Epidemiology and Public Health, Carlos III Health Institute, Madrid, Spain; 3 IMIM – Parc Salut Mar, Barcelona, Spain; 4 GRECS, Universitat de Girona, Girona, Spain; 5 Fundación Memora, Barcelona, Spain

## Abstract

People who have suffered from cancer find it difficult to return to work after a sickness absence (SA). Previous evidence indicates that people who survive a cancer have a higher risk than general population of leaving the labor market prematurely or being unemployed due to sequelae of both treatment and disease. Our objective is to identify barriers and facilitators associated with the return and permanence in the workplace of salaried workers after a SA due to cancer in Catalonia. The research used a descriptive qualitative approach with socio-constructivist perspective. A theoretical sampling was carried out until saturation. Three discussion groups (7 people/group) were conducted with people who had suffered a SA due to cancer in Catalonia. The sessions were held virtually and were recorded, transcribed verbatim, and analyzed using thematic analysis and mixed coding with Atlas.ti. Most of the people had returned to work after SA or were looking for a job that was suitable for their health status. Among the barriers to reincorporation to their job detected: (1) coping with the same workload they had before the SA, (2) sequelae associated with cancer treatment that affected their ability to work (stress, low ability to concentrate, chronic fatigue, mobility limitations), (3) having jobs with a high physical load, (4) expectations of colleagues and bosses. Among the facilitating factors: (1) sessions with psycho-oncologists, (2) availability of holidays to adapt their return after SA, (3) teleworking, and (4) job adaptation. Regarding proposals to improve this process, the most outstanding were the implementation of policies that allow a gradual return to work adjusted to the people who want to adhere to it and generalize the possibility of doing psycho-oncological therapy. End of SA after cancer is a key moment for people who go through it, they suffer many difficulties during the process that could be prevented with measures such as a gradual return to work.

**Key messages:**

• Workers who suffer a SA due to cancer face difficulties on their return to work.

• Initiatives of adaptation and gradual reincorporation to the workplace could improve return to work process after a SA due to cancer.

